# Multi-Omics Analysis Elucidates Flavor Evolution and Bioformation Mechanisms of Key Aroma Compounds in Malty-Aroma Yogurt

**DOI:** 10.3390/foods15020272

**Published:** 2026-01-12

**Authors:** Zihao Liu, Qihao Wang, Shiheng Luo, Chen Xing, Wenlu Li, Hong Zeng, Yanbo Wang

**Affiliations:** 1 School of Food and Health, Beijing Technology and Business University, Beijing 100048, China; 2330201009@st.btbu.edu.cn (Z.L.); 2531031037@st.btbu.edu.cn (Q.W.); 2330202076@st.btbu.edu.cn (C.X.); 20230908@btbu.edu.cn (W.L.); 2 Key Laboratory of Geriatric Nutrition and Health, Ministry of Education, Beijing Technology and Business University, Beijing 100048, China; 3 Shimadzu Co., Ltd., China Innovation Center, Beijing 100020, China; spklsh@shimadzu.com.cn

**Keywords:** *Lactococcus lactis*, time-series flavoromics, time-series metabolomics, correlation network analysis, precise flavor modulation

## Abstract

Flavor enrichment in commercial yogurts commonly relies on exogenous flavoring agents, failing to meet consumer demand for clean-label products with natural ingredients. This study developed a starter culture for malty-aroma yogurt by combining *Lactococcus lactis* BL-19 with a commercial yogurt starter culture. Evaluation by eleven trained sensory assessors indicated that malty-aroma yogurt exhibited a distinctive sensory profile compared with four commercially available plain yogurts. Time-series flavoromics identified 13 key aroma compounds and revealed the 3 h as the key time node of flavor evolution during fermentation. Furthermore, time-series metabolomics analysis revealed metabolic transitions from nutrient adaptation to active biosynthesis at 3 h, significantly increasing the odor-active values of the key aroma compounds. Moreover, correlation network analyses revealed potential metabolic precursors and metabolic bypass associated with the production of key aroma compounds and highlighted the valine, leucine and isoleucine biosynthesis pathway as central to malty-aroma formation. This study elucidates the evolution of flavor compounds and the underlying bioformation mechanisms of malty-aroma yogurts, offering insights for the precise flavor modulation of fermented dairy products.

## 1. Introduction

Yogurt is one of the most widely consumed fermented dairy products in the world and is valued for its nutritional density and favorable sensory attributes [1]. The global yogurt market continues to expand steadily, and China accounts for a substantial share of both consumption and revenue [2]. Streptococcus thermophilus (ST) and *Lactobacillus delbrueckii* subsp. *bulgaricus* (LB) are the most commonly used starter cultures for yogurt fermentation, interacting synergistically to generate secondary metabolites that shape the characteristic flavor profile [3]. Although more than 117 volatile odor compounds have been identified in yogurt, only 31 have been confirmed as aroma-active by gas chromatography-olfactometry–mass spectrometry (GC-O-MS) [4]. Recent shifts in consumer attitudes have accelerated the demand for clean-label foods, characterized by short ingredient lists and recognizable, minimally processed components. However, traditional yogurt fermentation typically results in inherently mild flavors. To increase sensory perception and meet consumer expectations, manufacturers frequently add flavoring agents, which may compromise product authenticity and consumer trust [5]. Although consumers increasingly claim a preference for clean label products, actual purchasing decisions are often still influenced by flavor, taste, and aroma [6]. Moreover, to ensure consistent fermentation across diverse processing conditions, commercial yogurts are often produced using standardized starter cultures. While beneficial for manufacturing control, this can result in subdued aroma and homogenized sensory characteristics, ultimately reducing consumer appeal and purchase intent [7].

A malty aroma is an appreciated sensory attribute and a hallmark of fermented beverages such as beer, primarily originating from malt-based compounds [8]. *Lactococcus lactis* subsp. *lactis* BL-19 (*L. lactic* BL-19) rapidly produces lactic acid and is noted for high 3-methylbutanol production, which imparts a distinctive malty aroma [9]. Previous studies have demonstrated that the malt- and nut-like aroma of 3-methylbutanol is increasingly preferred by consumers and positively influences purchase intention [10,11]. Gas chromatography-mass spectrometry (GC-MS) is commonly used to analyze the volatile compounds in yogurt by separating and identifying molecules based on their mass spectral signatures. For instance, it has been used to investigate the impact of *Ganoderma lucidum* water extract on the volatile compound profile of set-type yogurt [12]. Despite the growing interest in the flavor characteristics of yogurt, most studies only examine the final product, while minimal research is available on the dynamic evolution of volatile compounds during fermentation. Consequently, the key biochemical transitions that contribute to flavor formation remain unclear.

Volatile flavor compounds are typically end products of complex microbial metabolism. Although they directly shape sensory perception, their temporal dynamic profiling alone is insufficient to systematically elucidate the underlying mechanisms of flavor development during fermentation. Metabolomics techniques are currently an effective approach for investigating the dynamic small-molecule metabolite changes in yogurt [13]. It enables the characterization of a broad range of metabolites, including amino acids and lipids, providing critical insights into the metabolic state of the yogurt matrix at specific fermentation stages. Many of these metabolites serve as biosynthetic precursors of volatile aroma compounds. They also participate in the enzymatic and biochemical transformations responsible for flavor formation. Therefore, integrating metabolomics and flavoromics helps elucidate the underlying bioformation mechanisms during the flavor development process.

This study developed a starter culture by combining *L. lactis* BL-19 and a commercial yogurt starter culture to obtain a yogurt with a malty aroma. The fermentation process was monitored by measuring the pH, viable cell counts, lactic acid concentration, and microbial diversity. The dynamic changes in volatile aroma compounds and non-volatile metabolites during fermentation were characterized using time-series GC-MS and ultra-high performance liquid chromatography-tandem time-of-flight mass spectrometry (LC-QTOF-MS). Furthermore, a correlation-based metabolic network linking key metabolites and aroma compounds was constructed to investigate the metabolic routes responsible for the characteristic malty aroma formation.

This study aims to enhance flavor modulation during fermentation in the dairy industry while also expanding the aroma diversity and potential applications of aroma-producing lactic acid bacteria in commercially available yogurt. Meanwhile, a general analytical platform integrating time-series flavoromics and metabolomics with correlation-based network analysis was established, providing a systematic framework for the precise regulation of flavor in fermented dairy products.

## 2. Materials and Methods

### 2.1. Materials and Reagents

The *L. lactis* BL-19 (CGMCC No. 16242) was obtained from InnerMongolia Agricultural University, while the Yoflex Premium 5.0 yogurt starter culture was purchased from Chr. Hansen (Hørsholm, Denmark). The whole milk powder was sourced from Fonterra (Auckland, New Zealand), and sucrose was obtained from Fuzheng Donghai (Guangzhou, China). Four different types of flavored yogurt were purchased from a local supermarket. The M17 medium was supplied by Beijing Land Bridge Technology Co., Ltd. (Beijing, China). The methanol, acetonitrile, dichloromethane, and 2-methyl-3-heptanone were purchased from Aladdin (Shanghai, China).

### 2.2. Preparation of the Malty-Aroma Yogurt

The reconstituted milk was prepared using a previously delineated method with slight modifications [14]. Briefly, 11.5% (*wt*/*wt*) whole milk powder and 6.5% (*wt*/*wt*) sucrose were dissolved in distilled water at 50 °C and mixed with a magnetic stirrer (IKA) for 15 min to ensure complete dissolution. The mixture was homogenized at 20 MPa using a high-pressure homogenizer (Duoning AH-NANO, Shanghai, China) and pasteurized at 95 °C for 5 min. All post-pasteurization operations were conducted in aseptic conditions on a clean bench. The activated *L. lactis* BL-19 was inoculated into the reconstituted milk cooled to 37 °C, ensuring a final concentration of 1 × 10^6^ CFU/mL, followed by fermentation at 37 °C. Notably, in commercial stirred yogurt products, added sugars are commonly used to enhance sweetness and balance acidity, with total sugar levels often higher than those applied in experimental fermentation systems, and sugar addition is frequently conducted after fermentation rather than prior to fermentation (as applied in this study) to allow better control of the final sweet–sour balance [15]. The addition of sucrose in this study was intended to provide a nutritionally supportive and standardized fermentation matrix [16], rather than to replicate the sweetness profile of a finished commercial product.

To evaluate the fermentation characteristics of the malty-aroma yogurt, the pH dynamics were monitored using a pH meter and the lactic acid content was quantified since these parameters reflected the acidification capacity and directly affected the final product quality. The viable cell counts were also determined to assess the changes in microbial proliferation throughout the fermentation process. To examine the impact of *Lactococcus lactis* BL-19 supplementation, the same analyses were performed on a control yogurt fermented solely with the commercial starter culture.

### 2.3. Quantitative Descriptive Analysis

Quantitative descriptive analysis (QDA) was conducted to examine the impact of BL-19 addition on fermented yogurt flavor. This assessment included single BL-19-fermented, malty-aroma yogurt, and four commercially available yogurts with varying flavor profiles [17], and was performed in accordance with ISO 8586:2012 [18]. The sensory panel consisted of 11 trained assessors (five males and six females). Each panelist had over two years of experience in the sensory evaluation of dairy products and demonstrated the ability to identify common yogurt aromas such as sour, milky, buttery, and creamy notes.

After group discussion, six sensory attributes were selected to best represent the flavor profiles of the tested yogurts: fermented, milky, creamy, cheesy, buttery, and malty aromas. Three individual samples (10–15 mL each) were prepared in transparent tasting cups for each flavor type and labeled with randomized three-digit codes. A ten-point intensity scale was used for scoring, where 0 indicated no perception, 7 represented the reference intensity, and 9 denoted a much stronger-than-reference perception. The sensory evaluations were performed at room temperature (24 ± 2 °C), with a 30 s interval between samples, during which coffee beans were provided to help restore olfactory sensitivity. The final score for each sample was calculated as the average of the three evaluations.

### 2.4. Determination of the Fermentation Phenotype

Fermentation phenotype, including the pH level, lactic acid content, and viable cell count, were determined to monitor the proliferation of the malty-aroma yogurt starter culture in milk. Reconstituted milk inoculated with the BL-19 and commercial starter culture was placed in a thermostatic water bath at 37 °C for fermentation, with sampling at 1 h intervals throughout the process in aseptic conditions on a clean bench. The plate dilution coating method was used to determine the viable cell count, while a pH meter (FiveEasy Plus, Mettler Toledo, Zurich, Switzerland) was employed to measure the pH level and a biosensor analyzer SBA-40ES (YanHe Biotechnology, Weifang, China) was utilized to assess the lactic acid content. Before lactic acid analysis, the yogurt samples were serially diluted to adjust the lactic acid concentration to within the detectable range of the instrument (0–50 mg/100 mL). For each measurement, 10 μL of the diluted sample was transferred into the SBA-40ES analyzer, after which the lactic acid concentration in the yogurt was determined by multiplying the instrument reading by the corresponding dilution factor [19].

### 2.5. 16S rRNA Sequencing

The bacterial 16S rRNA amplicon sequencing was performed by Majorbio Bio-Pharm Technology Co., Ltd. (Shanghai, China), while the total microbial DNA was extracted using a E.Z.N.A.^®^ soil DNA Kit (Omega Bio-tek, Norcross, GA, USA) according to the instructions of the manufacturer. The 338F/806R primers were employed to amplify the V3–V4 region of the 16S rRNA gene using a T100 Thermal Cycler (Bio-Rad, Hercules, CA, USA). The purified amplicons were pooled in equimolar ratios and sequenced on an Illumina NextSeq 2000 platform (Illumina, San Diego, CA, USA). The raw sequencing reads were deposited into the NCBI SRA database and processed using fastp for quality filtering and FLASH for merging (mismatch ratio ≤ 0.2). The sequences were clustered into OTUs with 97% similarity using UPARSE 7.1, while taxonomy was assigned via RDP Classifier (confidence threshold 0.7) against the Silva v138 database. The quantitative OTU abundance was determined using the standard curves of the spike-in DNA copy numbers.

### 2.6. HS-SPME Extraction Procedure and GC-MS Analysis

#### 2.6.1. HS-SPME Extraction

The flavor compounds of yogurt were extracted using SPME-Arrow (Shimadzu Smart SPME Arrow 1.10 mm: DVB/C-WR/PDMS, Kyoto, Japan). A 5 g yogurt sample and 0.5 g NaCl were transferred to a 20 mL headspace vial with a silicone septum (JianqiangWeiye, Beijing, China) and vortexed to enhance extraction efficiency. Next, 1 µL of 2-methyl-3-heptanone was added at a concentration of 0.816 mg/mL as an internal standard and incubated at 45 °C for 30 min, after which the SPME-Arrow fiber was exposed to the headspace at 45 °C for 30 min.

#### 2.6.2. GC-MS Analysis

A GCMS-TQ8050 NX system (Shimadzu Corporation, Kyoto, Japan) was combined with the Shimadzu Smart Aroma Database for GC-MS analysis. An SH-polar wax capillary column (60 m × 0.25 mm, 0.25 µm) was employed for chromatographic separation, using helium (99.999%) as the carrier gas at a flow rate of 1.0 mL/min. The initial column temperature of 40 °C was maintained for 5 min, then increased to 200 °C at a rate of 3 °C/min. The SPME-Arrow fiber was inserted into the injection port in splitless mode at 250 °C and desorbed for 5 min. Before sample extraction, the fiber was conditioned in the injection port for 5 min to remove potential contaminants. The mass spectrometry conditions included electron ionization (EI) at 70 eV, with an ion source temperature of 200 °C and a mass scanning range of 35–600 m/z in full scan mode [20].

The volatile compounds in the yogurt were qualitatively analyzed by matching against the NIST 20 mass spectral library (https://chemdata.nist.gov/), calculating the retention index (RI) of compounds relative to a series of n-alkanes (C7–C40), and comparing the calculated RI values with those reported in the literature. The internal standard method was applied to quantify the volatile compounds according to the peak ratio of each compound to that of the internal standard.

### 2.7. Metabolomics Analysis via LC-QTOF-MS

#### 2.7.1. Sample Preparation

Metabolite extraction was performed using a method described by Zhang et al. [21], with slight modifications. The metabolite extraction solvent was prepared by mixing methanol, acetonitrile, and water at a ratio of 2:2:1 (*v*/*v*/*v*). Frozen yogurt samples (0.4 g) were thawed at room temperature (24 ± 2 °C) and mixed with 4 mL of the extraction solvent, vortexed thoroughly and ultrasonicated three times below 4 °C for 10 min each. The mixture was left to stand at −20 °C for 10 min and centrifuged at 14,000× *g* for 20 min at 4 °C. The supernatant was collected and evaporated to dryness under a nitrogen stream. Before LC-MS analysis, the dried extracts were reconstituted in acetonitrile/water (1:1, *v*/*v*), while the quality control (QC) samples were prepared by pooling equal volumes of the extracts from each specimen.

#### 2.7.2. LC-QTOF-MS Analysis

The metabolomics analysis was performed using a UPLC-Triple TOF 6600 system (AB SCIEX) with an ACQUITY UPLC BEH Amide column (2.1 mm × 100 mm, 1.7 µm; Waters, Wexford, Ireland). Mobile phase A consisted of 25 mM ammonium acetate and 25 mM ammonium hydroxide in water, while mobile phase B comprised acetonitrile. The other parameters included a column temperature of 25 °C, a flow rate of 0.5 mL/min, and an injection volume of 2 μL. The gradient elution program included 5% A and 95% B from 0 to 0.5 min, a linear B decrease from 95% to 65% from 0.5 to 6.5 min, a linear B decrease from 65% to 40% from 6.5 to 7.5 min, where it was maintained for 1 min. B was increased to 95% within 0.1 min, followed by a 3 min re-equilibration period.

The samples were analyzed in both positive and negative electrospray ionization (ESI) modes, both at spray voltage of ±5500 V and an ion source temperature of 600 °C. The mass spectrometry conditions included a mass scanning range of 60–1000 Da for MS1 and 25–1000 Da for MS2, and respective accumulation times of 0.20 s and 0.05 s per spectrum [22].

The raw MS data were converted to MzXML files using ProteoWizard MSConvert before being imported into freely available XCMS software (Version 3.7.1). Collection of Algorithms of MEtabolite pRofile Annotation (CAMERA) was sued for isotope and adduct annotation. The metabolite compounds were identified by comparing the accuracy of the *m*/*z* value (<10 ppm) and MS/MS spectra with an in-house database established with available authentic standards.

### 2.8. Statistical Analysis

The samples were analyzed in triplicate, and the results were presented as mean ± standard deviation. The statistical analysis of variance and significance testing were performed using one-way ANOVA followed by Duncan’s multiple range test (*p* < 0.05). SIMCA was used for principal component analysis (PCA), partial least squares discriminant analysis (PLS-DA), and calculation of the variable importance in projection (VIP) values. The heatmaps were generated using TBtools v2.310, while MetaboAnalyst: https://dev.metaboanalyst.ca/MetaboAnalyst/ (accessed on 27 May 2025.) was employed for pathway enrichment analysis. All other figures were created using Origin (version 2021, OriginLab, Hampton, MA, USA).

## 3. Results

### 3.1. Fermentation Phenotype

BL-19 addition increased the initial viable cell count at 0 h and reduced the fermentation duration from 6.5 h to 6 h. The lactic acid concentrations in the malty-aroma yogurt consistently exceeded those in the control at all fermentation stages, correlating with the faster fermentation rate. However, except for the first hour, the viable cell counts in the malty-aroma yogurt remained lower than those of the control.

When co-cultured with commercial starter cultures, the system exhibited metabolic changes that differed from those observed during cultivation with the commercial starters alone, possibly due to cross-feeding interactions between BL-19 and *ST*. Specifically, *ST* can effectively consume lactose and rapidly acidify milk, while *L. lactis* can utilize galactose not fully metabolized by *ST* [23]. In addition, a clear cross-feeding relationship was evident between leucine and adenine in the *ST* and *L. lactis* co-culture model [24]. Furthermore, *L. lactis* often shows a higher capacity for aroma compound production in co-culture conditions, which may indicate a lower biomass accumulation rate but higher aromatic compound synthesis efficiency [25].

### 3.2. Microbial Diversity

**Figure 1:**
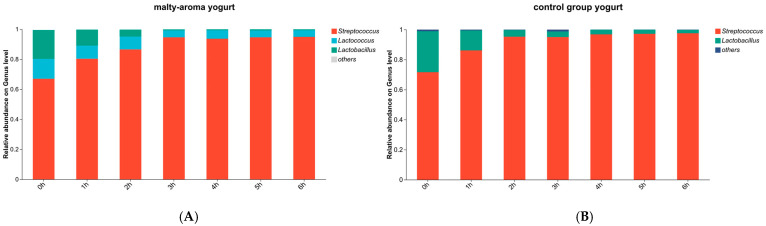
The 16S rRNA sequencing of the yogurt starter culture. (**A**) The malty-aroma yogurt. (**B**) The control-group yogurt.

Samples were collected immediately after inoculation to determine the initial microbial composition and examine the relative abundance of each fermentation strain. Additional samples were collected from the malty-aroma yogurt and the control group throughout fermentation, as described in Section 2.4. Three bacterial genera were identified: *Streptococcus*, *Lactobacillus*, and *Lactococcus*. *Streptococcus* and *Lactobacillus* originated from Chr. Hansen Yoflex Premium 5.0, corresponding to *ST* and *LB*, respectively, whereas *Lactococcus* was derived from *L. lactis* BL-19 (Figure 1).

Consistent with typical yogurt fermentation profiles, *Streptococcus* was the most abundant genus, with *ST* dominating throughout the process [26], with the malty-aroma yogurt exhibiting a similar trend. At the time of inoculation, *ST* accounted for 63% of the microbial community, while its relative abundance exceeded 90% by 6 h. Contrarily, the relative abundance of the *LB* in the control group declined gradually during fermentation, which was consistent with previous reports [27,28]. The *ST* exhibited a selective growth advantage [29], making it dominant in diverse yogurt types. It grew rapidly during the early fermentation stage by consuming free amino acids and subsequently promoted *LB* growth by producing formic acid [30]. The relative abundance of *L. lactis* BL-19 decreased during fermentation, eventually stabilizing at approximately 4%, which likely favored the development of the malty yogurt aroma.

### 3.3. Sensory Evaluation

**Figure 2:**
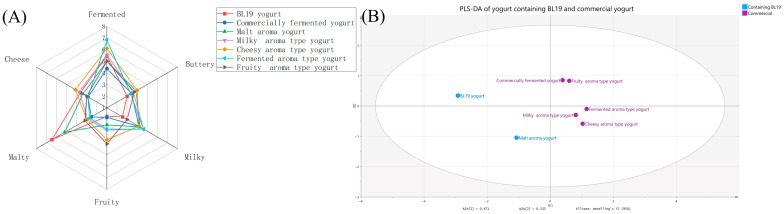
The QDA of the yogurt. (**A**) The radar chart. (**B**) The PLS-DA score plots showing sample discrimination based on sensory profiles.

QDA was performed to determine whether the incorporation of BL-19 enhanced the malty aroma in yogurt compared to that fermented solely with the commercial starter. It also assessed whether adding the commercial starter compensated for the reduced intensity of other aroma attributes typically observed in yogurt fermented with BL-19 alone. Therefore, BL-19-fermented yogurt and yogurt produced with the commercial starter were used as the primary controls for evaluating the malty-aroma yogurt. The examination focused on four plain yogurt flavors, including fermented, cheesy, milky, and fruity aromas. The malty aroma stood out as the most distinctive attribute for differentiating between the yogurt types. This was expected since malty is not a typical descriptor for yogurt [31,32], and is more often associated with products such as beer and cheese. Conversely, the BL-19-fermented yogurt showed relatively low levels of several characteristic yogurt aromas, including milky, fruity, and buttery attributes. However, adding the commercial starter to the malty-aroma yogurt increased or balanced these aroma perceptions. These results indicated that combining BL-19 with a commercial culture not only imparted a distinct malty note but also restored broader sensory complexity (Figure 2A).

The PLS-DA plot of the flavor compounds in the yogurts with different aromas (Supplementary Information) further corroborated the trends observed in the QDA results. Specifically, 3-methylbutanol was identified as a major contributor to the higher malty sensory scores in the malty-aroma and BL-19 yogurts compared with the other samples. Contrarily, compounds with fruity-like odors, including 2-butanone, 2-octanone, and butanoic acid, showed clustering and were positioned away from the malty-aroma yogurt and the BL-19 yogurt in the PLS-DA space. This separation helped explain why these two yogurts exhibited a slightly weaker fruity aroma intensity than the other samples.

Regarding the flavored yogurts, except for the milky-flavor sample, each type showed the expected dominance in its labeled aroma attribute. This likely reflected the shared aroma characteristics of these products and the inherent difficulty in independently distinguishing and quantifying individual components within complex milky odors [33]. These findings were further supported by the loading plots of the different aroma types, where common milky-aroma compounds, such as 2,3-butanedione and acetone, were located near the center of the loading diagrams, indicating their minimal contribution to distinguishing between the yogurt aroma types (Figure 2B).

### 3.4. Volatile Compounds Profiles

**Figure 3:**
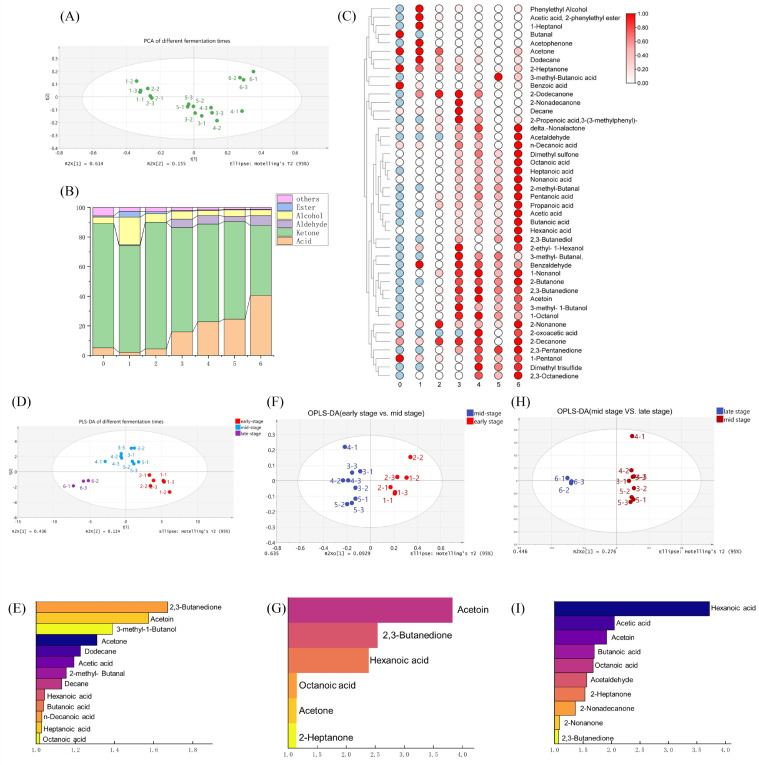
The changes in the volatile compounds during the fermentation of the malty-aroma yogurt. (**A**) The PCA score plot. (**B**) The bar chart of the total concentrations of the different types of volatile compounds. (**C**) The hierarchical clustering heatmap. (**D**) The PLS-DA of the different fermentation stages. (**E**) The VIP plot of PLS-DA. (**F**,**H**) The OPLS-DA plots for the different fermentation stages. (**G**,**I**) The VIP plots of OPLS-DA.

Clear differences were evident between the sensory attributes and volatile flavor compounds of the malty-aroma yogurt and the commercially available yogurts with various aroma profiles. The dynamic changes in volatile compounds during fermentation were analyzed by combining SPME-Arrow and GC-MS to investigate the formation of the distinctive malty aroma. This approach identified 44 volatile compounds during the 6 h fermentation process, including 13 acids, five aldehydes, eight alcohols, 12 ketones, and six other compounds (Figure 3). However, the concentration of a compound alone did not directly reflect its aroma intensity, as different compounds exhibited markedly different odor detection thresholds. For instance, diacetyl, with a low detection threshold of 0.2 ng/g, is easily produced and represents a characteristic flavor compound in dairy products. To quantify the sensory contribution of the volatile compounds, the odor activity value (OAV) was calculated as the ratio of the concentration of a compound to its perception threshold. An OAV < 1 indicated that the compound was not perceivable, while an OAV ≥ 1 suggested it might contribute to the aroma. Table 1 summarizes the odor-active compounds identified during the monitoring of dynamic volatile flavor formation. A total of 18 compounds with OAV ≥ 1 were identified during the malty-aroma yogurt fermentation, of which 13 were still detected after the 6 h fermentation process and were considered the key aroma compounds. Of these, ketones were the most abundant, yielding six compounds, followed by three aldehydes (acetaldehyde, 2-methylbutanal, and 3-methylbutanal) and two acids (acetic acid and hexanoic acid). Alcohols and other compound types were represented by one compound each. Notably, 3-methylbutanal (malty) and 3-methylbutanol (malty), with OAVs of 3 and 7, contributed substantially to the characteristic malty aroma. Furthermore, typical yogurt flavor compounds such as acetaldehyde, diacetyl, and acetoin exhibited high OAVs of 42, 93, and 29, respectively. These intense baseline aroma contributors helped explain why malty-aroma yogurt exhibited stronger milky, fruity, and buttery sensory notes compared with yogurt fermented solely with *L. lactis* BL-19.

PCA, an unsupervised dimensionality-reduction method [34], was used to summarize the flavoromics data into two principal components (Figure 3A). The PCA results suggested clear stage-dependent separation across the different fermentation stages. PC1 and PC2 denoted 61.4% and 15.5% of the total variance, respectively, accounting for 76.9% cumulatively. This indicated that PCA effectively distinguished between the flavor characteristics in the samples. The clustering patterns reflected consistent volatile profiles and high reproducibility within each group. The samples at 1–2 h were mapped to the upper-left region, while those at 3–5 h clustered near the center. The 6 h samples formed a separate cluster in the upper-right region, illustrating the progressive shift in flavor composition during fermentation. This was consistent with previous studies showing that raw milk generally exhibited a relatively bland flavor profile [35], while the incorporation of yogurt starter cultures markedly enhanced flavor development [36]. In the present study, as fermentation progressed, the malty-aroma yogurt starter culture gradually transformed the milk with a low abundance of aroma-active compounds (five aroma compounds with OAV ≥ 1) into a malty-aroma yogurt characterized by a considerably higher number of aroma-active compounds (13 aroma compounds with OAV ≥ 1).

The PCA score plot suggested that the 6 h fermentation process was divided into three distinct phases. The VIP scores derived via PLS-DA were used to quantify the discriminative power of the individual metabolites for classifying these phases [37] (Figure 3D). Based on VIP ≥ 1, 13 volatile flavor compounds were identified as belonging to distinct fermentation stages, including three ketones (2,3-butanedione, acetoin, and acetone), six acids (acetic, hexanoic, butanoic, decanoic, heptanoic, and octanoic acid), two alkanes (dodecane and decane), 3-methylbutanol, and 2-methylbutanal. Short-chain acids (C2–C4) were primarily produced by lactic acid bacteria during fermentation, while long-chain acids (C4–C20) were mainly derived from milk fat degradation [38]. As the fermentation progressed, the lactic acid bacteria metabolized the milk nutrients, which promoted growth and proliferation. This metabolism produced lactic acid, which acidified the milk, as well as acetic acid and long-chain acids (e.g., hexanoic acid). These compounds contributed floral, fruity, and cheesy aromas, explaining the gradual increase in acid concentrations over time (Figure 3B). The 2,3-butanedione and acetoin ketones dominated in the yogurt and imparted buttery and creamy notes, representing key contributors to the characteristic flavor [20]. Their concentrations increased significantly during mid-to-late fermentation, enriching the flavor profile of the malty-aroma yogurt. Contrarily, acetone, a common source of off-flavor in yogurt [39], was the only volatile compound with a VIP > 1 that showed a significant decrease as fermentation progressed. Although starter cultures may produce small amounts of acetone during growth, this substance is typically derived from raw milk [40].

Furthermore, 3-methylbutanol and 3-methylbutanal contributed significantly to the distinctive flavor of the malty-aroma yogurt. These compounds are synthesized via the Ehrlich pathway through leucine degradation. Both compounds are commonly present in heat-processed milk products [41] and cheese [42], significantly impacting the aroma due to their low odor detection thresholds of 0.15 ng/g and 4 ng/g, respectively. However, these substances are often not regarded as key aroma contributors in yogurt [43] since the malty aroma is not considered a typical yogurt flavor. This is primarily because *ST* mainly supplies formate, folate, and carbon dioxide during fermentation to stimulate *LB* growth, while relying on *LB*-mediated proteolysis through the PrtB membrane-bound proteinase [44]. Because most *ST* strains exhibit weak intrinsic proteolytic activity, their cooperation with *LB* largely depends on this cross-feeding mechanism. However, only non-proteolytic (Prt−) *ST* establish stable interactions with *LB* since proteolytic *ST* (Prt+) incur additional metabolic costs associated with proteinase production that reduce their suitability [45]. Consequently, Prt− strains grow faster and are more likely to become dominant during fermentation [46]. This ecological advantage of Prt− *ST* also explains the observation that the introduction of proteolytic *L. lactis* BL-19 reduced the viable counts throughout fermentation in the malty-aroma yogurt compared with the control. In addition to the inability of dominant *ST* strains to hydrolyze proteins efficiently, most starter cultures lack the downstream enzyme systems required for converting leucine into 3-methylbutanal. Specifically, leucine must first be transformed into α-ketoisocaproate via transaminases and then decarboxylated by branched-chain keto acid decarboxylase (KdcA) to form 3-methylbutanal, which is subsequently reduced to 3-methylbutanol [47]. However, most lactic acid bacteria lack the gene encoding KdcA [48], which is why these two malt-aroma compounds are rarely present in commercially available yogurts. Additionally, 2-methylbutanol, an aroma compound with a nutty note perceptually similar to malty flavors, was produced during the same fermentation stage as 3-methylbutanol. By the end of fermentation, 2-methylbutanol showed an OAV > 7, indicating a substantial contribution to the aromatic complexity and richness of the malty-aroma yogurt.

Orthogonal partial least squares discriminant analysis (OPLS-DA) was used for the pairwise comparisons between the fermentation stages to clarify the volatile compound differences (Figure 3F–I). Six volatiles were identified that discriminated between the early and mid-fermentation phases, including four ketones (acetoin, 2,3-butanedione, acetone, and 2-heptanone) and two acids (hexanoic and octanoic acid), representing key yogurt flavor contributors. Furthermore, 10 volatiles were detected that distinguished between mid and late fermentation, including four acids (hexanoic, acetic, butanoic, and octanoic acid), five ketones (acetoin, 2-heptanone, 2-nonadecanone, 2-nonanone, and 2,3-butanedione), and acetaldehyde. Acetaldehyde is a hallmark yogurt flavor compound [49], typically perceived as fresh and fruity at low concentrations [50]. It is widely believed to be produced in large quantities during the late stage of yogurt fermentation [40,51]. However, the data from this experiment revealed gradual acetaldehyde formation as early as the mid-fermentation (3 h). This substance accumulated rapidly from the mid- to late stages, peaking at the end.

Overall, 3 h of fermentation was a critical time point for flavor development in the malty-aroma yogurt, showing a considerable increase in the total volatile compound concentrations (Figure 3B). Notably, five of the 13 key aroma compounds became odor-active from this time onward, including 3-methylbutanal, the compound responsible for the malty aroma. Moreover, acetaldehyde, a characteristic flavor compound of yogurt, was detected for the first time at 3 h. These results indicated that the characteristic malty aroma of the yogurt started to develop gradually at 3 h.

### 3.5. Metabolomics Analysis

LC-MS-based metabolomics is a key approach for studying small-molecule metabolites [52]. It enables metabolite identification in yogurt and mapping to metabolic pathways, providing insight into the effect of treatment on product composition and quality [1,53]. The yogurt samples obtained throughout fermentation were subjected to metabolomics analysis using liquid chromatography–quadrupole time-of-flight mass spectrometry (LC-QTOF-MS) to elucidate malty aroma formation and identify the putative volatile compound precursors.

#### 3.5.1. PCA and Differential Metabolite Identification

**Figure 4:**
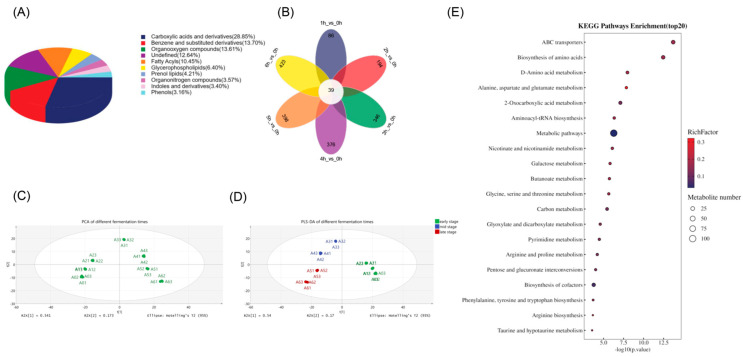
The metabolomics analysis of the malty-aroma yogurt during fermentation. (**A**) The proportions of the different types of metabolites identified in positive and negative ion modes. (**B**) A Venn diagram of the different fermentation times. (**C**) The PCA plots of the different fermentation times. (**D**) The PLS-DA plots of the different fermentation stages. (**E**) The KEGG enrichment analysis presented as a bubble plot.

A total of 1354 metabolites were identified in both positive and negative ion modes. The most abundant categories included carboxylic acids and derivatives (28.85%), benzene and substituted derivatives (13.70%), organooxygen compounds (13.61%), fatty acyls (10.45%), and glycerophospholipids (6.40%). As fermentation progressed, the number of differential metabolites increased across the stages (Figure 4B). The PCA score plot (Figure 4C) indicated good biological reproducibility and reliable differential metabolite patterns. Clear separation was observed among the samples collected at different fermentation times, demonstrating that fermentation duration strongly influenced the global metabolic profiles. The first two principal components accounted for 54.1% (PC1) and 17.3% (PC2) of the total variance, demonstrating that the main metabolic differences among the samples were primarily due to temporal variation during fermentation. PCA further revealed a clear time-dependent trajectory: early-stage samples (0–3 h) were positioned on the right side of the plot, mid-stage samples (3–4 h) were clustered in the third quadrant, and late-stage samples (5–6 h) were grouped in the second quadrant. This stage-specific separation highlighted pronounced metabolic transitions throughout the fermentation process. Notably, this temporal pattern differed from that observed for volatile compounds, indicating that metabolite profile transitions and aroma-related changes did not occur simultaneously.

This study used *p* < 0.05 and VIP > 1 as parameters to identify 604 differential metabolites in both positive and negative ion modes. The heatmap of the differential metabolites (Figure S1) showed a similar pattern: the 0–2 h samples formed one cluster, while the 3–4 h and 5–6 h samples were grouped together into a larger separate cluster. This could be attributed to the comprehensive metabolite coverage afforded by metabolomic analysis, which enabled the identification of diverse non-volatile metabolites that function as precursors of volatile aroma compounds. In the microbially driven fermentation system, these precursor metabolites were continuously converted via enzyme-catalyzed reactions and microbial metabolism, resulting in dynamic changes over time and shaping the evolution of the aroma profile [54]. Notably, both volatile-compound and metabolomics analyses showed significant changes at 3 h, which was subsequently identified as a key time point in malty-aroma yogurt fermentation. Consistent with previous studies [55], the observed metabolic transitions might be attributed to the coordinated enzymatic degradation, transformation, and biosynthesis associated with amino acid metabolism, lipid turnover, and carbohydrate utilization.

#### 3.5.2. Pathway and Enrichment Analysis of the Differential Metabolites

The metabolites were mapped to KEGG, followed by pathway enrichment analysis to characterize the metabolic changes throughout the fermentation of the malty-aroma yogurt. The differential metabolites mapped to pathways were primarily clustered in the metabolism category, which was further subdivided into amino acid metabolism (10 pathways), carbohydrate metabolism (10 pathways), global and overview maps (seven pathways), cofactor and vitamin metabolism (five pathways), energy metabolism (three pathways), nucleotide metabolism (one pathway), and lipid metabolism (one pathway) (Figure 4E). Of these, carbohydrate and amino acid metabolism represented the dominant categories.

The metabolism of carbohydrates, such as fructose, mannose, and galactose, is pivotal during fermentation [56]. This study showed notable galactose metabolism enrichment since it could be converted into glucose-6-phosphate through a series of enzymatic steps, subsequently entering the glycolysis pathway to supply energy. Beyond its role as a carbon source, galactose also affected the protein glycosylation levels [57], necessitating its inclusion in yogurt starter strains [58]. Amino acid biosynthesis and metabolism were critical during lactic acid bacteria fermentation and served as major precursors for many volatile and non-volatile flavor compounds. The enrichment of the arginine biosynthesis pathway suggested enhanced nitrogen metabolism flux during fermentation. This facilitated the conversion of nitrogen metabolites into intermediates of the tricarboxylic acid (TCA) cycle, contributing to energy metabolism. This was consistent with the accumulation of citrate, pyruvate, and L-glutamate in the alanine, aspartate, and glutamate metabolism pathway. Furthermore, alanine decreased by 0.8-fold at the end of fermentation. Since alanine metabolism is crucial for cell wall synthesis and cellular defense, its decline may indicate active utilization by the microbial community to support cell wall construction and adaptation to an increasingly acidic environment. The levels of differential metabolites enriched in pathways related to key aroma compounds fluctuated significantly as fermentation progressed. Throughout the fermentation process, the leucine and ketoleucine concentrations changed by 0.72- and 10.45-fold, respectively, in the valine, leucine, and isoleucine biosynthesis pathway. These compounds served as substrates and direct precursors for the formation of 3-methylbutanal, a key aroma compound responsible for the malty aroma. These findings indicated that carbohydrate and amino acid metabolism were primarily responsible for flavor profile formation during malty-aroma yogurt fermentation.

#### 3.5.3. Metabolic Transitions at the Key Time Point

**Figure 5:**
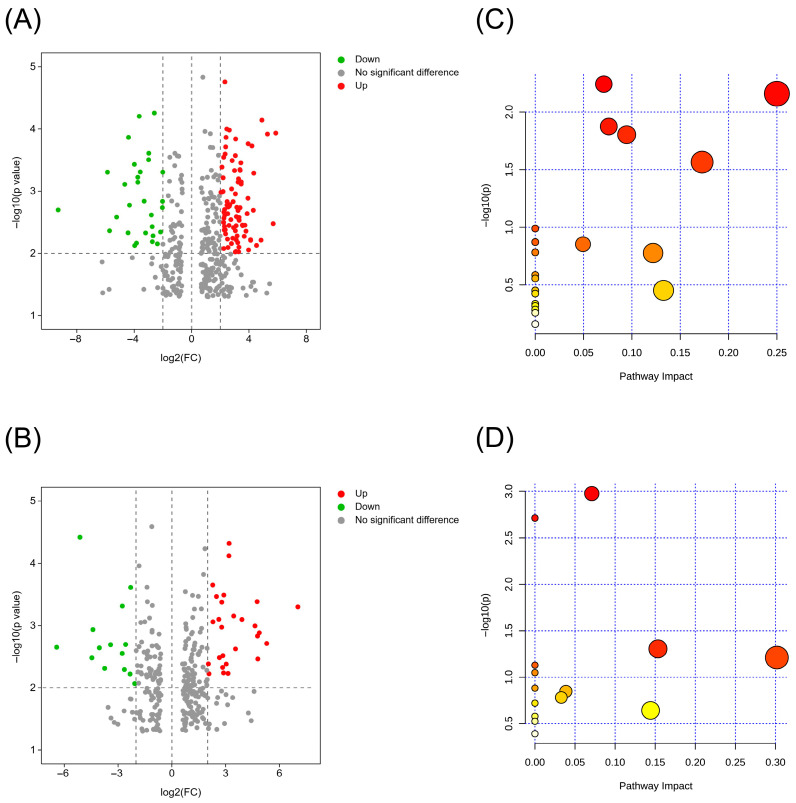
The metabolomics analysis of the key time point. (**A**,**B**) The volcano plots for 0 h vs. 3 h and 3 h vs. 6 h, respectively. (**C**,**D**) The KEGG enrichment analysis for 0 h vs. 3 h and 3 h vs. 6 h, respectively.

This study compared metabolite changes between the 0–3 h and 3–6 h fermentation stages to elucidate why the 3 h mark was a critical time point. A total of 385 and 350 differential metabolites were identified in the 0 h vs. 3 h and 3 h vs. 6 h comparisons, respectively. The large transition in metabolite profiles within the first 3 h reflected the rapid initiation of metabolic activity as fermentation began, while the slightly lower number of differential metabolites between 3 h and 6 h suggested a metabolic pattern stabilization during sustained logarithmic growth. The volcano plots (Figure 5A,B), constructed with |Log_2_Fold change| > 2 and *p* < 0.01, illustrated these stage-dependent differences. Pathway enrichment analysis of these metabolites provided more compelling evidence, revealing that 160 metabolites that changed significantly between 0 h and 3 h were associated with 24 pathways, which primarily converged into three modules directly relevant to flavor formation. The carbohydrate metabolism pathways, including galactose, amino-sugar, and nucleotide-sugar metabolism, and O-antigen nucleotide-sugar biosynthesis, were significantly activated. The pyruvate, L-lactic acid, and pyruvaldehyde levels increased 34-, 37-, and 6.57-fold, respectively. This indicated a substantial acceleration in carbon flux toward lactic acid production, which denoted initial milk sugar conversion into lactic acid, representing a key factor in yogurt sourness and one of the earliest detectable sensory changes [59]. Furthermore, the glucose 1-phosphate, UDP-galactose, and UDP-N-acetylglucosamine levels increased 6.18-, 4.84-, and 4.77-fold, respectively, indicating enhanced milk carbohydrate conversion into intermediate substrates that supported both energy supply and the subsequent biosynthesis of aroma-related molecules [60].

Protein metabolism pathway enrichment revealed the strong activation of amino acid biosynthesis, supporting the escalating demand for building blocks during accelerated microbial proliferation. These pathways not only facilitated biomass formation but also modulated the availability of aroma precursors. The phenylalanine, tyrosine, tryptophan, valine, leucine, and isoleucine biosynthesis pathways were significantly enriched before 3 h. D-amino acid metabolism emerged as a significant substance during the initial 3 h period, showing a high pathway relevance score. D-amino acids are involved in cell wall remodeling and competition-driven proteolysis, while their turnover is increasingly recognized as an important contributor to flavor development [61]. The enhanced metabolism of these chiral amino acid derivatives suggests that microbial adaptation to acidifying conditions may inadvertently promote the release of short peptides and aroma precursors that participate in subsequent aroma compound formation [62]. During nucleotide metabolism, the pyrimidine metabolism pathway was markedly activated in the 0–3 h period, reflecting the substantial nucleotide demand associated with rapid DNA/RNA synthesis in logarithmic growth. While not directly involved in flavor formation, this metabolic activation expanded the enzymatic capacity (e.g., proteases, aminotransferases, and decarboxylases), enabling subsequent amino acid catabolism that drove key aroma compound generation.

During the 3–6 h phase, 64 significantly altered metabolites (|Log_2_FC| > 2, *p* < 0.01) were enriched in 16 pathways, of which 11 were amino acid-related. The enhanced degradation of the milk proteins and peptide turnover during amino acid catabolism reflected both sustained biosynthetic demand and intensified competition between the strains. As described in Section 3.4, this stage also represented the period during which many key aroma compounds in the malty-aroma yogurt began to exhibit odor activity, denoting the gradual development of the overall flavor profile.

These findings indicated that the first 3 h marked a metabolic transition from nutrient adaptation to active biosynthesis that established precursors of both the taste-determining organic acids and key aroma-forming molecules. During this stage, metabolic flux was primarily directed toward carbohydrate breakdown and amino acid precursor generation, providing a foundation for sour-taste formation and subsequent aroma synthesis. From 3 h to 6 h, the system entered a distinct metabolic phase characterized by accelerated precursor conversion into volatile flavor compounds. The fatty acid catabolism, amino acid transamination, and ester biosynthesis pathways became increasingly dominant, presenting fruity and malty aroma notes that began to define the sensory profile of malty-aroma yogurt. Therefore, 3 h represented a biologically meaningful metabolic node that signaled the transition from precursor accumulation to aroma production, integrating taste development and aroma onset into continuous flavor formation in the malty-aroma yogurt. This also explained the considerable aroma compound concentration increase observed in Section 3.4.

### 3.6. Correlation Network Analysis

**Figure 6:**
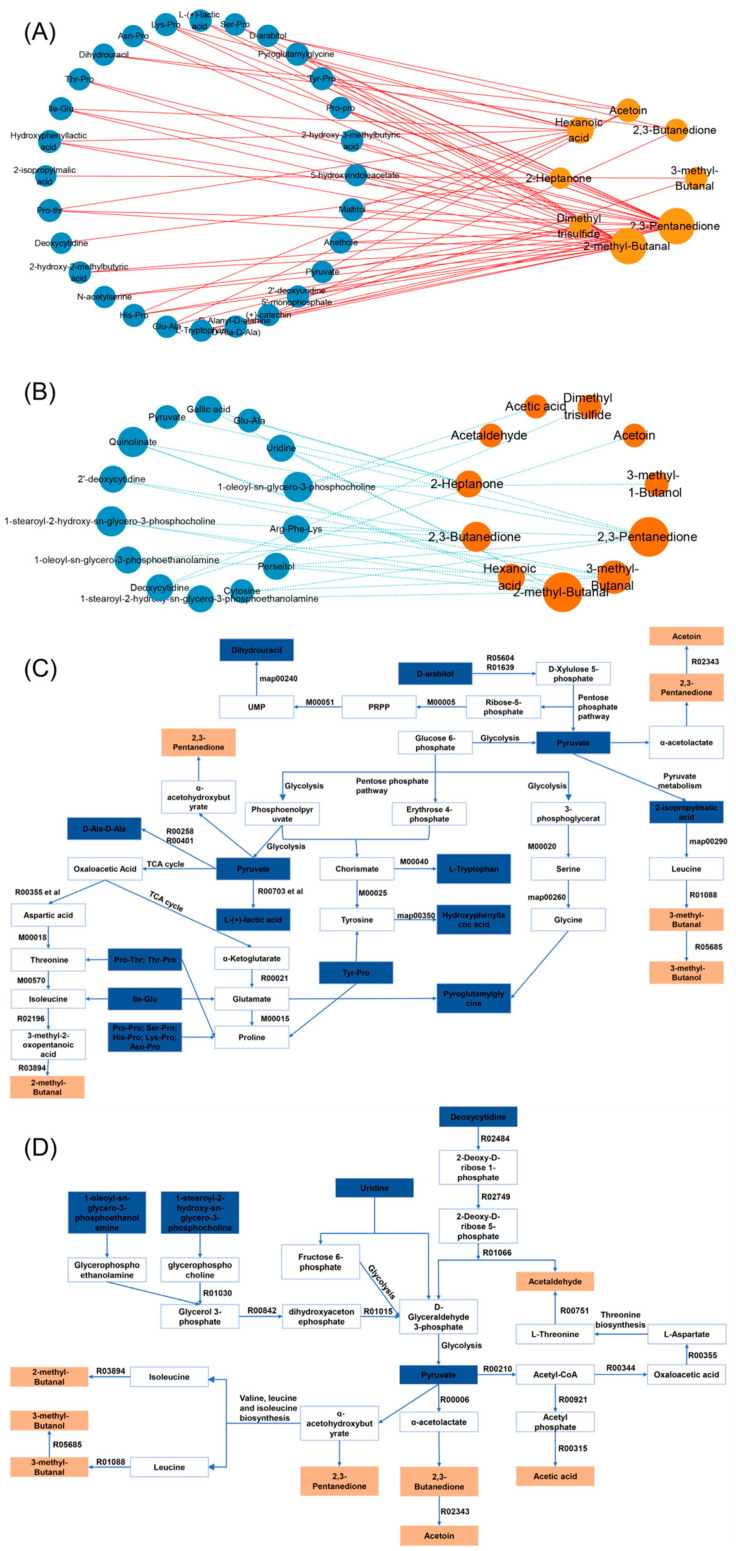
The correlation network analysis. The (**A**) positive and (**B**) negative differential metabolite correlations with the key aroma compounds (|r| > 0.9). The orange nodes represent volatile flavor compounds, and blue nodes denote non-volatile metabolites. The solid red lines indicate positive correlations, while the blue dashed lines denote negative correlations. The size of each circle reflects the number of metabolites associated with each compound. The (**C**) positive and (**D**) negative correlations between the metabolic networks of the key aroma compounds and the differential metabolites. The orange nodes represent volatile aroma compounds, while blue nodes denote the differential metabolites exhibiting significant correlations.

A Spearman correlation analysis was performed to investigate the relationship between the volatile and non-volatile compounds during the malty-aroma yogurt fermentation (Figure 6). A total of 11 aroma compounds and 39 metabolites with VIP > 2 and *p* < 0.01 yielded 90 significant correlations (|r| > 0.9, *p* < 0.05), of which 68.89% (62 correlations) were positive.

Several lipid-derived metabolites, including 1-stearoyl-2-hydroxy-sn-glycero-3-phosphocholine and 1-stearoyl-2-hydroxy-sn-glycero-3-phosphoethanolamine, showed a strong negative association with 2,3-pentanedione and hexanoic acid. This suggested a sequential metabolic process in which the lactic acid bacteria first degraded the milk fat and generated intermediate or precursor compounds during yogurt fermentation. As the fermentation progressed, these precursors were further converted into 2,3-pentanedione and hexanoic acid, causing rapid accumulation after 3 h. Similarly, negative associations between the 3-methylbutanal and peptide-derived metabolites indicated that leucine catabolism gradually transitioned from biomass synthesis to aroma generation during the mid-to-late fermentation stage. These negative correlations supported the interpretation that the formation of key aroma compounds was accompanied by continuous consumption of their biosynthetic precursors.

Most of the significant correlations related to 2-methylbutanal were positive (21 in total), indicating a metabolic rerouting involving small peptides, amino acids, and their derivatives. This suggested that central carbon-metabolism intermediates were either allocated to anabolic processes, such as cellular structures and protein turnover, or diverted toward branched-chain amino acid (BCAA) catabolism, which drove the formation of nutty and malty aroma compounds.

Figure 6C,D present the metabolic network in which the key aroma compounds are connected to their strongly correlated metabolites (|r| > 0.9) via multistep biochemical transformations. These correlated metabolites mainly included amino acids and their derivatives, such as 3-methylbutanal, 3-methylbutanol, and 2-methylbutanal, as well as carbohydrate metabolism intermediates, including 2,3-butanedione, acetoin, and acetaldehyde. The network was constructed using KEGG pathway annotations for *Lactococcus lactis* subsp. *lactis* and was further refined with the previously reported biosynthetic routes for flavor-related metabolites. Notably, a negative association was evident between many amino acid-derived metabolites and key aroma aldehydes, likely representing upstream precursors that were depleted as fermentation progressed, directing them into aroma-forming pathways. This depletion pattern was consistent with the multistep enzymatic conversion of branched-chain amino acids to aldehydes and then to alcohols, as evidenced by the KEGG-based pathway network (Figure 6D).

Contrarily, the positive correlations between certain aroma compounds and peptide- or nucleotide-related metabolites suggested the presence of bypass fluxes originating from shared intermediates. This redirected the metabolic flow either toward biosynthetic routes that supported cellular growth and maintenance or toward catabolic branches that generated aroma-active aldehydes and alcohols. Therefore, the simultaneous presence of positive and negative correlations highlighted a dual metabolic control mechanism in which precursor depletion drove aroma formation, while bypass redistribution modulated the overall flux balance between growth and flavor production.

Of the affected pathways identified in Section 3.5.3, the valine, leucine, and isoleucine biosynthesis pathway had the most significant impact on the development of the malty yogurt aroma. In this study, pyruvate was directed into this pathway (map00290) via condensation reactions catalyzed by acetohydroxyacid synthase. Two pyruvate molecules formed α-acetolactate, the potential precursor of valine and leucine, while the reaction between pyruvate and 2-ketobutyrate produced α-acetohydroxybutyrate, the precursor of isoleucine.

The subsequent catabolism of these branched-chain amino acids contributed directly to key aroma compound formation. Leucine degradation yielded 3-methylbutanal, a characteristic malty note, which was further reduced to 3-methylbutanol with similar sensory attributes. Similarly, 2-methylbutanal, associated with a nutty aroma, resulted from isoleucine degradation. The strong correlations between these key aroma compounds and peptide- or amino acid-related metabolites suggested metabolic flux diverged from shared precursors, which were redirected either for biomass synthesis and cellular maintenance or toward catabolic pathways that generated aroma-active aldehydes and alcohols.

This metabolic network highlighted how central metabolic processes were channeled toward flavor formation, with pyruvate emerging as a pivotal hub that connected most key aroma compounds and their strongly correlated metabolites (|r| > 0.9) and linked amino acid metabolism with carbohydrate-derived aroma formation pathways. This central role of pyruvate is consistent with recent findings showing that it serves as the immediate precursor of lactic acid and other organic acids produced during fermentation [63]. Moreover, pyruvate metabolism is pivotal during yogurt production, governing both flavor compound generation and acidity development [64]. As shown in Figure 6D, the differential metabolites showing significant negative correlations with key aroma compounds acted as potential precursors that yielded the corresponding flavor substances via multistep enzymatic reactions. Furthermore, pyruvate served as an important intermediate linking energy, lipid, and nucleotide metabolism, facilitating the production of multiple flavor compounds, such as acetaldehyde, 2,3-butanedione, 2,3-pentanedione, and acetoin, via their respective precursors.

These findings indicated that aroma formation was governed by both flux rerouting and cooperative conversion within central metabolic pathways. Therefore, strategically modifying the flow of potential metabolic bypasses to prioritize the production of key aroma compounds may effectively enhance malty aroma formation in fermented dairy products.

## 4. Conclusions

This study developed a malty-aroma yogurt starter culture by co-cultivating a malty-aroma-producing *Lactococcus lactis* strain with a commercial yogurt starter. Its distinctive flavor profile was validated using QDA, which compared it with four commercially available plain yogurts. A total of 18 odor-active compounds were identified during malty-aroma yogurt fermentation, of which 13 remained by the end of the process. These substances were considered key aroma compounds and included acetaldehyde, 2-methylbutanal, 3-methylbutanal, 2,3-Butanedione, 2,3-pentanedione, 2-heptanone, 3-methylbutanol, acetoin, dimethyl trisulfide, 2-nonanone, acetic acid, 2-decanone, and hexanoic acid. Time-series flavoromics analysis indicated that the 3 h fermentation mark was critical for malty aroma formation. The flavor compound concentrations increased considerably at this point, while five of the 13 key aroma compounds achieved OAV > 1 for the first time. Time-series metabolomic analysis further revealed that carbohydrate and amino acid metabolism represented the main pathways responsible for the overall flavor profile of malty-aroma yogurt. In addition, a shift from nutrient adaptation to active biosynthesis was evident at 3 h, markedly increasing the flavor compound levels. Time-series flavoromics and metabolomics were combined with correlation network analysis to elucidate the dynamic flavor compound evolution and the associated bioformation mechanisms in malty-aroma yogurt, providing valuable insights for precise flavor modulation.

This study highlighted the practical applications of aroma-producing lactic acid bacteria in fermented dairy products. The integrated workflow that combined time-series flavoromics and metabolomics and correlation-based network analysis yielded a generalizable platform for identifying the flavor of aroma-producing LAB, helping to clarify their bioformation mechanisms in dairy matrices. In particular, identifying 3 h of fermentation as a key flavor-evolution node provides a measurable and operational reference point linking microbial metabolic transitions to the onset of odor-active compounds. This information can guide the selection of specific strains or co-cultures and optimize fermentation conditions to achieve desired aroma profiles. From an industrial perspective, malty-aroma yogurt represents a distinct and previously underexplored aroma type in yogurt production. This can enhance the flavor diversity in fermented dairy products while not violating the clean-label expectations. Notably, it is vital to clarify that the correlation network aims to objectively prioritize candidate precursors and metabolic pathways, rather than establish direct causality. Therefore, further validation in pilot or industrial-scale settings will be essential for confirming these findings.

## Figures and Tables

**Table 1:** The odor thresholds and changes in the OAVs of the odor-active compounds during the fermentation of the malty-aroma yogurt.

No.	Compound	Odor Threshold in Water (ng/g) ^a^	Aroma Descriptor	OAV ^b^						
				0	1	2	3	4	5	6
2	acetaldehyde	2.7	Fresh	-	-	-	25	28	8	42
3	acetone	40	Sweet	1	1	1	0.43	0.56	0.46	0.91
5	2-methylbutanal	2.2	Nutty	-	-	-	3	4	4	7
6	butanal	2	Aldehydic	8.14	-	-	-	-	-	-
7	3-methylbutanal	0.15	Malty	-	-	-	18	11	12	3
8	2,3-Butanedione	2	Creamy	-	2	15	101	112	74	93
9	decane	6.6	Waxy	0.80	0.78	0.72	2	1	0.42	0.56
10	2,3-pentanedione	20	Buttery	-	-	-	1	1	1	2
12	2-heptanone	10	Fruity	30	26	22	22	16	17	21
13	3-methylbutanol	4	Malty	-	0.53	1	10	5	4	7
15	acetoin	14	Creamy	-	0.22	1	28	38	19	29
17	dimethyl trisulfide	0.035	Sulfurous	-	-	-	-	34	21	30
18	1-heptanol	5.4	Fatty	-	8	0.43	0.12	0.25	-	-
19	2-nonanone	5	Fruity	4	0.41	9	4	5	2	10
21	acetic acid	124	Sour	-	-	-	0.24	0.28	0.18	1
26	2-decanone	3	Fruity	2	2	2	2	2	1	3
33	2-dodecanone	1.4	Waxy	-	0.75	1	1	0.82	-	-
36	hexanoic acid	65	Cheesy	0.14	0.08	0.06	1		2	7

^a^: Odor thresholds were referenced as a published book, named: Odor thresholds-compilations *of* odor threshold values in air, water and other media *(2nd ed*). ^b^: Odor activity values (OAV) were calculated by dividing the internal standard quantitative concentrations by the odor thresholds. -: Not detected.

## Data Availability

The original contributions presented in this study are included in the article/Supplementary Materials. Further inquiries can be directed to the corresponding authors.

## Supplementary Materials

The following supporting information can be downloaded at: https://www.mdpi.com/article/10.3390/foods15020272/s1.

## References

[1] Xue R., Liu J., Zhang M., Aziz T., Felemban S., Khowdiary M.M., Yang Z. (2024). Physicochemical, microbiological and metabolomics changes in yogurt supplemented with lactosucrose. Food Res. Int..

[2] Chen B., Zhang X., Zhou Q. (2021). Product differentiation and brand building: A hedonic analysis of yogurt price in China. Int. Food Agribus. Manag. Rev..

[3] Zhou X., Wang H., Wang C., Tan Q., Liu Y., Chen H., Zhang Y., Zhang Y., Liu S., Suo H. (2024). Improvement of brown yogurt quality by Lactiplantibacillus plantarum S58 and oat β-glucan: Physicochemical properties, sensory quality, and metabolic changes. Food Biosci..

[4] Liu C., Yang P., Wang H., Song H. (2022). Identification of odor compounds and odor-active compounds of yogurt using DHS, SPME, SAFE, and SBSE/GC-O-MS. LWT.

[5] Maruyama S., Streletskaya N.A., Lim J. (2021). Clean label: Why this ingredient but not that one?. Food Qual. Prefer..

[6] Yeretzian C., Pollien P., Lindinger C., Ali S. (2004). Individualization of Flavor Preferences: Toward a Consumer-centric and Individualized Aroma Science. Compr. Rev. Food Sci. Food Saf..

[7] Celik O.F., Temiz H. (2022). Lactobacilli isolates as potential aroma producer starter cultures: Effects on the chemical, physical, microbial, and sensory properties of yogurt. Food Biosci..

[8] Su X., Yu M., Wu S., Ma M., Su H., Guo F., Bian Q., Du T. (2022). Sensory lexicon and aroma volatiles analysis of brewing malt. Npj Sci. Food.

[9] Li W., Ren M., Duo L., Li J., Wang S., Sun Y., Li M., Ren W., Hou Q., Yu J. (2020). Fermentation Characteristics of Lactococcus lactis subsp. lactis Isolated From Naturally Fermented Dairy Products and Screening of Potential Starter Isolates. Front. Microbiol..

[10] Chen C., Tian T., Yu H., Yuan H., Wang B., Xu Z., Tian H. (2024). Aroma-sensory properties of Gouda cheeses based on young Chinese consumers’ preferences. J. Dairy Sci..

[11] Zhang X.Y., Guo H.Y., Zhao L., Sun W.F., Zeng S.S., Lu X.M., Cao X., Ren F.Z. (2011). Sensory profile and Beijing youth preference of seven cheese varieties. Food Qual. Prefer..

[12] Bao C., Yan M., Diao M., Anastasiia U., Zhang X., Zhang T. (2025). Effect of Ganoderma lucidum water extract on flavor volatiles and quality characteristics of set-type yogurt. Food Chem..

[13] Zhang R., Jia W. (2023). Brown goat yogurt: Metabolomics, peptidomics, and sensory changes during production. J. Dairy Sci..

[14] Yu X., Sun Y., Shen X., Li W., Cai H., Guo S., Sun Z. (2024). Effect of different isolation sources of Lactococcus lactis subsp. lactis on volatile metabolites in fermented milk. Food Chem. X.

[15] O’Rell K., Chandan R.C. (2013). Manufacture of various types of yogurt. Manufacturing Yogurt and Fermented Milks.

[16] Wang J., Guo Z., Zhang Q., Yan L., Chen Y., Chen X.I.A., Liu X.-M., Chen W.E.I., Zhang H.-P. (2010). Effect of probiotic Lactobacillus casei Zhang on fermentation characteristics of set yogurt. Int. J. Dairy Technol..

[17] Qiu S., Han H., Zeng H., Wang B. (2024). Machine learning based classification of yogurt aroma types with flavoromics. Food Chem..

[18] (2012). Sensory Analysis—General Guidelines for the Selection, Training and Monitoring of Selected Assessors and Expert Sensory Assessors.

[19] Yang L.-H., Liu M.-z., Chen Z.-L., Tong L.-L., Guo D.-S. (2024). Lipidomic and transcriptomic analysis of the increase in eicosapentaenoic acid under cobalamin deficiency of *Schizochytrium* sp.. Biotechnol. J..

[20] Han H., Zhang Z., Yang Z., Blank I., Zhong F., Wang B., Wang Y., Zeng H. (2024). A comparative study to determine the key aroma components of yogurt aroma types based on Sensomics and Flavoromics. Food Chem..

[21] Zhang X., Zheng Y., Liu Z., Su M., Wu Z., Zhang H., Zhang C., Xu X. (2024). Insights into characteristic metabolites and potential bioactive peptides profiles of fresh cheese fermented with three novel probiotics based metabolomics and peptidomics. Food Chem. X.

[22] Chen X., Zhu Z., Zhang X., Chen L., Gu Q., Li P. (2024). Lactobacillus paracasei ZFM54 alters the metabolomic profiles of yogurt and the co-fermented yogurt improves the gut microecology of human adults. J. Dairy Sci..

[23] Lugli Gabriele A., Argentini C., Tarracchini C., Longhi G., Mancabelli L., Bianchi Massimiliano G., Taurino G., Amaretti A., Candeliere F., Bussolati O. (2025). Host interactions of Lactococcus lactis and Streptococcus thermophilus support their adaptation to the human gut microbiota. Appl. Environ. Microbiol..

[24] Özcan E., Seven M., Şirin B., Çakır T., Nikerel E., Teusink B., Toksoy Öner E. (2021). Dynamic co-culture metabolic models reveal the fermentation dynamics, metabolic capacities and interplays of cheese starter cultures. Biotechnol. Bioeng..

[25] Canon F., Maillard M.-B., Henry G., Thierry A., Gagnaire V. (2021). Positive Interactions between Lactic Acid Bacteria Promoted by Nitrogen-Based Nutritional Dependencies. Appl. Environ. Microbiol..

[26] Suh S.H., Kim M.K. (2021). Microbial communities related to sensory characteristics of commercial drinkable yogurt products in Korea. Innov. Food Sci. Emerg. Technol..

[27] Luzzi G., Brinks E., Fritsche J., Franz C.M.A.P. (2020). Microbial composition of sweetness-enhanced yoghurt during fermentation and storage. AMB Express.

[28] Kochetkova T.V., Grabarnik I.P., Klyukina A.A., Zayulina K.S., Elizarov I.M., Shestakova O.O., Gavirova L.A., Malysheva A.D., Shcherbakova P.A., Barkhutova D.D. (2022). Microbial Communities of Artisanal Fermented Milk Products from Russia. Microorganisms.

[29] Ben-Yahia L., Mayeur C., Rul F., Thomas M. (2012). Growth advantage of Streptococcus thermophilus over Lactobacillus bulgaricus in vitro and in the gastrointestinal tract of gnotobiotic rats. Benef. Microbes.

[30] Qiu S., Zeng H., Yang Z., Hung W.-L., Wang B., Yang A. (2023). Dynamic metagenome-scale metabolic modeling of a yogurt bacterial community. Biotechnol. Bioeng..

[31] Majchrzak D., Lahm B., DÜRrschmid K. (2010). Conventional and Probiotic Yogurts Differ in Sensory Properties but Not in Consumers’ Preferences. J. Sens. Stud..

[32] Zeng H., Han H., Huang Y., Wang B. (2023). Rapid prediction of the aroma type of plain yogurts via electronic nose combined with machine learning approaches. Food Biosci..

[33] Lawless H.T. (1999). Descriptive analysis of complex odors: Reality, model or illusion?. Food Qual. Prefer..

[34] Greenacre M., Groenen P.J.F., Hastie T., D’Enza A.I., Markos A., Tuzhilina E. (2022). Principal component analysis. Nat. Rev. Methods Primers.

[35] Lan L., Wang W., Su Y., Xu H., Han J., Chi X., Xi Y., Sun B., Ai N. (2025). Exploration of milk flavor: From the perspective of raw milk, pasteurized milk, and UHT milk. Food Chem. X.

[36] Xu R., Tang L., Gao X., Han X., Liu C., Song H. (2025). Study of Aroma Characteristics and Establishment of Flavor Molecular Labels in Fermented Milks from Different Fermentation Strains. Foods.

[37] Dong X., Tian M., Liu M., Cheng M., Fang J., Zhao X., Li C., Guo W., Liu L. (2025). Multidimensional correlation analysis based on PCA/PLS-DA revealed the dual role of Lacticaseibacillus helveticus KLDS1.8701 in flavor synthesis and microbiota construction in Dongbei Suancai. J. Future Foods.

[38] Cheng H. (2010). Volatile flavor compounds in yogurt: A review. Crit. Rev. Food Sci. Nutr..

[39] Vaseghi Bakhshayesh R., Panahi B., Hejazi M.A., Nami Y. (2024). Metabolite profiling of different Iranian traditional yogurts using an untargeted metabolomics approach. Heliyon.

[40] Papaioannou G., Kosma I., Badeka A.V., Kontominas M.G. (2021). Profile of Volatile Compounds in Dessert Yogurts Prepared from Cow and Goat Milk, Using Different Starter Cultures and Probiotics. Foods.

[41] Wang B., Gao X., Song H., Sun H., Liu P., Liu A. (2025). Investigating the effects of sterilization temperatures on odor of high-temperature short-time pasteurized milk using molecular sensory science technique. Food Chem..

[42] Duensing P.W., Hinrichs J., Schieberle P. (2024). Influence of Milk Pasteurization on the Key Aroma Compounds in a 30 Weeks Ripened Pilot-Scale Gouda Cheese Elucidated by the Sensomics Approach. J. Agric. Food Chem..

[43] Chen C., Zhao S., Hao G., Yu H., Tian H., Zhao G. (2017). Role of lactic acid bacteria on the yogurt flavour: A review. Int. J. Food Prop..

[44] Sieuwerts S., Molenaar D., van Hijum Sacha A.F.T., Beerthuyzen M., Stevens Marc J.A., Janssen Patrick W.M., Ingham Colin J., de Bok Frank A.M., de Vos Willem M., van Hylckama Vlieg Johan E.T. (2010). Mixed-Culture Transcriptome Analysis Reveals the Molecular Basis of Mixed-Culture Growth in Streptococcus thermophilus and Lactobacillus bulgaricus. Appl. Environ. Microbiol..

[45] Settachaimongkon S., Nout M.J.R., Antunes Fernandes E.C., Hettinga K.A., Vervoort J.M., van Hooijdonk T.C.M., Zwietering M.H., Smid E.J., van Valenberg H.J.F. (2014). Influence of different proteolytic strains of Streptococcus thermophilus in co-culture with Lactobacillus delbrueckii subsp. bulgaricus on the metabolite profile of set-yoghurt. Int. J. Food Microbiol..

[46] Sieuwerts S., de Bok Frank A.M., Hugenholtz J., van Hylckama Vlieg Johan E.T. (2008). Unraveling Microbial Interactions in Food Fermentations: From Classical to Genomics Approaches. Appl. Environ. Microbiol..

[47] Clark S., Clark S., Drake M., Kaylegian K. (2023). Cheddar and Cheddar-Type Cheeses. The Sensory Evaluation of Dairy Products.

[48] Smit B.A., Engels W.J.M., Smit G. (2009). Branched chain aldehydes: Production and breakdown pathways and relevance for flavour in foods. Appl. Microbiol. Biotechnol..

[49] Karagül-Yüceer Y., Drake M. (2013). Sensory analysis of yogurt. Manufacturing Yogurt and Fermented Milks.

[50] Li D., Cui Y., Wu X., Li J., Min F., Zhao T., Zhang J., Zhang J. (2024). Graduate Student Literature Review: Network of flavor compounds formation and influence factors in yogurt. J. Dairy Sci..

[51] Zhao X., Ge Y., Yu X., Liu C., Li H., Wang X., Yao S. (2024). Fermentation Characteristics of Fermented Milk with Streptococcus thermophilus CICC 6063 and Lactobacillus helveticus CICC 6064 and Volatile Compound Dynamic Profiles during Fermentation and Storage. Molecules.

[52] Wu W., Zhang L., Zheng X., Huang Q., Farag M.A., Zhu R., Zhao C. (2022). Emerging applications of metabolomics in food science and future trends. Food Chem. X.

[53] Yang S., Yan D., Zou Y., Mu D., Li X., Shi H., Luo X., Yang M., Yue X., Wu R. (2021). Fermentation temperature affects yogurt quality: A metabolomics study. Food Biosci..

[54] Park M.K., Kim Y.-S. (2021). Mass spectrometry based metabolomics approach on the elucidation of volatile metabolites formation in fermented foods: A mini review. Food Sci. Biotechnol..

[55] Jiang L., Chen Y., Zhao T., Li P., Liao L., Liu Y. (2025). Analysis of differential metabolites in Liuyang douchi at different fermentation stages based on untargeted metabolomics approach. Food Chem. X.

[56] Cheng S., Li W., Yang H., Hou B., Hung W., He J., Liang C., Li B., Jiang Y., Zhang Y. (2025). Integrated transcriptomics and metabolomics reveal changes during Streptococcus thermophilus JM66 fermentation in milk: Fermentation characteristics, flavor profile, and metabolic mechanism. Food Res. Int..

[57] Sakizli U., Takano T., Yoo S.K. (2024). GALDAR: A genetically encoded galactose sensor for visualizing sugar metabolism in vivo. PLoS Biol..

[58] Iskandar C.F., Cailliez-Grimal C., Borges F., Revol-Junelles A.-M. (2019). Review of lactose and galactose metabolism in Lactic Acid Bacteria dedicated to expert genomic annotation. Trends Food Sci. Technol..

[59] Zhang X., Zheng Y., Kumar Awasthi M., Zhou C., Barba F.J., Cai Z., Liu L., Rene E.R., Pan D., Cao J. (2022). Strategic thermosonication-mediated modulation of lactic acid bacteria acidification kinetics for enhanced (post)-fermentation performance. Bioresour. Technol..

[60] Wu Q., Cheung C.K.W., Shah N.P. (2015). Towards galactose accumulation in dairy foods fermented by conventional starter cultures: Challenges and strategies. Trends Food Sci. Technol..

[61] Roskjær A.B., Roager H.M., Dragsted L.O. (2024). D-Amino acids from foods and gut microbiota and their effects in health and disease. Food Rev. Int..

[62] Marcone G.L., Rosini E., Crespi E., Pollegioni L. (2020). D-amino acids in foods. Appl. Microbiol. Biotechnol..

[63] Liu N., Qin L., Mazhar M., Miao S. (2021). Integrative transcriptomic-proteomic analysis revealed the flavor formation mechanism and antioxidant activity in rice-acid inoculated with Lactobacillus paracasei and Kluyveromyces marxianus. J. Proteom..

[64] Yu G., Zhang C., Li X., Kong X., Chen Y., Hua Y. (2025). GC×GC-TOFMS and LC-ESI-MS/MS based flavoromics and metabolomics studies on the lactic acid bacteria fermentation of soymilks with dairy milk as the reference. Food Biosci..

