# Supplementary material for: Multi-Omics Analysis Elucidates Flavor Evolution and Bioformation Mechanisms of Key Aroma Compounds in Malty-Aroma Yogurt
Source: Foods. 2026 Jan 12;15(2):272. doi: 10.3390/foods15020272 (PMC12841482; doi:10.3390/foods15020272)

Figure S1. PCA plots and loading plots for different yogurt aroma types. (A) PCA plot. (B)loading diagrams. 1-7 represent cheesy and malty-aroma, milky, fruity; BL-19; commercial culture starter and fermented, respectively.

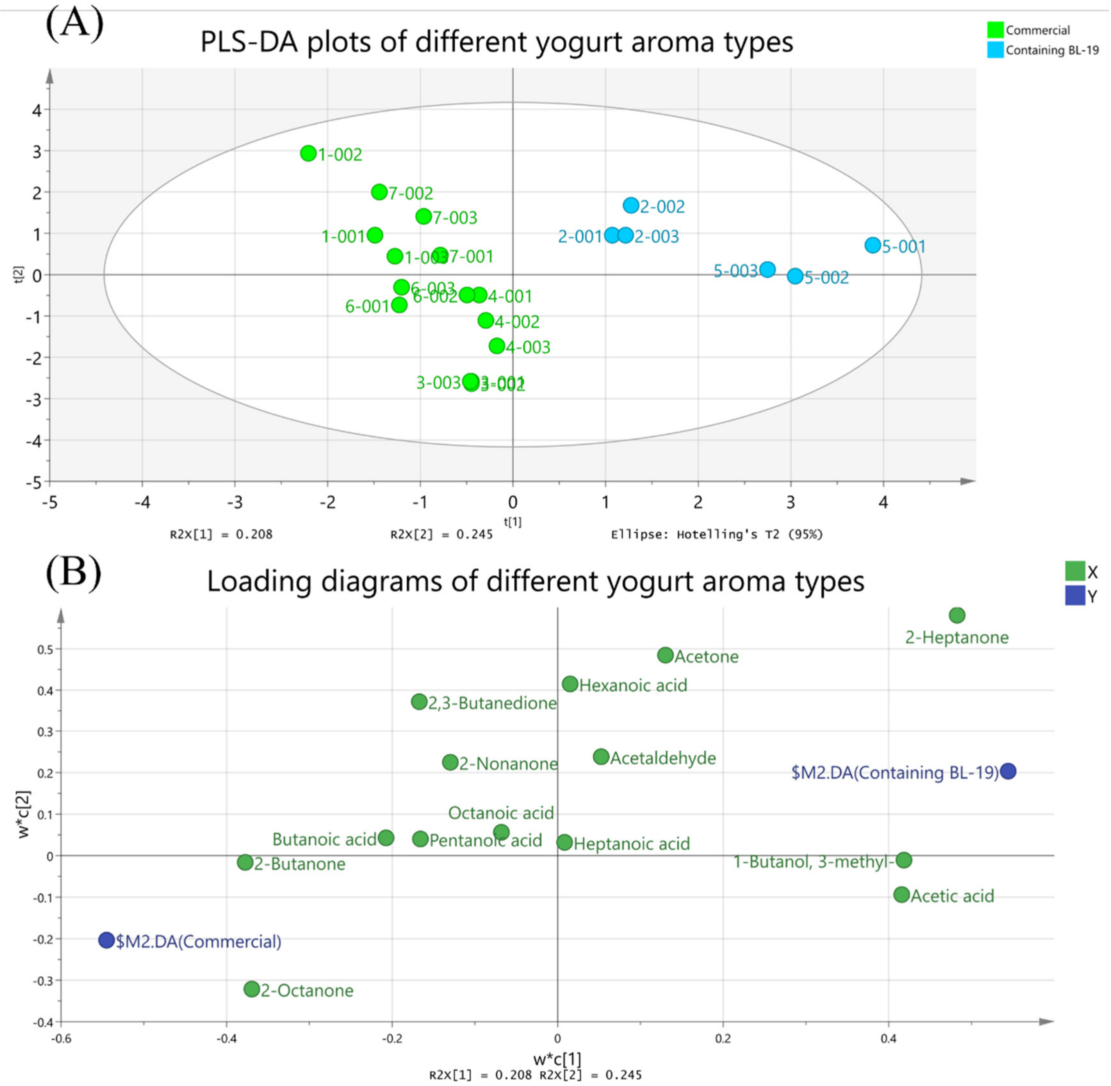

Table S1·Changes in the concentrations of flavor compounds at different fermentation times

No.	Compounds	0h	1h	2h	3h	4h	5h	6h
1	2-oxoacetic acid					0.00023±0.00003	0.00014±0.00002	0.00021±0.00001
2	Acetaldehyde				0.00696±0.00024	0.00774±0.00059	0.00233±0.00039	0.01157±0.00046
3	Acetone	0.00691±0.00	0.00712±0.00	0.00573±0.00	0.00174±0.00	0.00225±0.00	0.00183±0.00	0.00363±0.00

		224	372	354	043	104	074	08
4	2-Butanone				0.00213±0.00023	0.00222±0.00008	0.00166±0.00008	0.00271±0.00044
5	2-methyl-Butanal				0.00065±0.00016	0.00098±0.00011	0.00099±0.00023	0.00172±0.00018
6	Butanal	0.00163±0.0006						
7	3-methyl- Butanal,				0.00028±0.0001	0.00017±0.00005	0.00019±0.00002	0.00005±0.00001
8	2,3-Butanedione		0.00047±0.00028	0.003±0.00007	0.02029±0.00241	0.02245±0.00376	0.01497±0.00077	0.01874±0.00064
9	Decane	0.00053±0.00005	0.00051±0.00018	0.00048±0.00006	0.00175±0.00043	0.00082±0.00016	0.00028±0.00006	0.00037±0.00001
10	2,3-Pentanedione				0.00214±0.0003	0.00327±0.00027	0.00346±0.00008	0.00406±0.00052
11	Dodecane		0.00038±0.00009	0.00018±0.00003	0.00015±0.00002	0.00015±0.00001	0.00007±0.00001	0.00012±0.00001
12	2-Heptanone	0.03009±0.00187	0.02611±0.00528	0.02269±0.002	0.02273±0.00428	0.01663±0.01174	0.01774±0.00139	0.02112±0.0015
13	3-methyl- 1-Butanol		0.00021±0.00013	0.00054±0.00008	0.00413±0.00097	0.00219±0.00009	0.00196±0.00014	0.00292±0.0003
14	1-Pentanol	0.00203±0.00026	0.00131±0.00045	0.00117±0.00024	0.00101±0.00014	0.00182±0.00018	0.00141±0.00011	0.00181±0.00014
15	Acetoin		0.00031±0.00008	0.00184±0.00067	0.0404±0.00271	0.0536±0.01111	0.02752±0.00159	0.0406±0.00092
16	2,3-Butanediol				0.00046±0.00008		0.00051±0.00003	0.00091±0.00014
17	Dimethyl trisulfide					0.00012±0.00001	0.00008±0.00001	0.00011±0.00001
18	1-Heptanol		0.00466±0.00127	0.00023±0.00002	0.00007±0.00001	0.00013±0.00007		

19	2-Nonanone	0.00249±0.00131	0.0002±0.00005	0.00472±0.00026	0.0021±0.00198	0.00251±0.00229	0.00122±0.00137	0.00497±0.00017
20	2-Propenoic acid,3-(3-methylphenyl)-			0.00073±0.00005	0.00196±0.00017	0.0012±0.00015	0.00033±0.00009	0.00065±0.00007
21	Acetic acid				0.00301±0.00224	0.00342±0.00115	0.00224±0.00016	0.01242±0.00073
22	2-ethyl- 1-Hexanol		0.00022±0.0001		0.00065±0.00012	0±0		0.00056±0.00013
23	Benzaldehyde		0.00035±0.00006		0.00034±0.00003	0.00028±0.00003	0.00019±0	0.00021±0.00006
24	1-Octanol				0.00009±0.00001	0.0001±0.00003	0.00006±0.00001	0.00005±0.00002
25	Propanoic acid			0.00015±0.00004	0.00018±0.00008	0.00018±0	0.00009±0.00002	0.00041±0.00012
26	2-Decanone	0.00075±0.00008	0.00063±0.00013	0.00087±0.00004	0.00088±0.00003	0.00088±0.00011	0.00053±0.00005	0.00092±0.00012
27	Acetophenone		0.00018±0.00008					
28	Butanoic acid			0.00024±0.00003	0.00204±0.00067	0.0038±0.00061	0.00274±0.00019	0.00931±0.00054
29	1-Nonanol			0.00007±0.00005	0.00018±0.00001	0.00021±0.00007	0.0001±0.00001	0.0002±0.00001
30	3-methyl-Butanoic acid						0.0005±0.00008	0.00017±0.00009
31	2,3-Octanedione					0.00028±0.0001	0.00015±0.00003	0.00036±0.00005
32	Pentanoic acid			0.00016±0.00003	0.00028±0.00005	0.00051±0.0001	0.00037±0.00009	0.00079±0.00017
33	2-Dodecanone		0.00011±0.00002	0.00016±0.00001	0.00018±0.00006	0.00011±0.00005		

34	Acetic acid, 2-phenylethyl ester		0.0017±0.00094	0.00021±0.00006	0.00008±0.00002			
35	2-Nonadecanone				0.01061±0.00194		0.00007±0.00001	0.00016±0.00003
36	Hexanoic acid	0.00089±0.00028	0.00052±0.00026	0.00041±0.00016	0.01061±0.00194	0.02058±0.00369	0.01595±0.00208	0.04684±0.00478
37	Dimethyl sulfone	0.0006±0.00022	0.0006±0.00004	0.00066±0.00004	0.0011±0.00034	0.00157±0.00034	0.00099±0.00029	0.00203±0.00039
38	Phenylethyl Alcohol		0.00858±0.00669	0.00086±0.00014	0.00147±0.00043	0.00099±0.0004	0.00054±0.00017	0.00165±0.0002
39	Heptanoic acid				0.0002±0.00005	0.00023±0.00004	0.00015±0.00006	0.00045±0.00008
40	Benzoic acid	0.00087±0.00112	0.00015±0.0002			0.00017±0.00006	0.00005±0.00001	0.00009±0.00001
41	Octanoic acid	0.00045±0.00022	0.00032±0.0002	0.00029±0.00005	0.00422±0.00092	0.00483±0.00126	0.00277±0.00084	0.01039±0.00049
42	Nonanoic acid	0.00011±0.00004	0.00008±0.00005	0.00006±0	0.00047±0.00015	0.00063±0.00011	0.00041±0.00011	0.00116±0.00025
43	delta.-Nonalactone	0.00039±0.00006	0.00043±0.00006	0.00044±0.00001	0.00055±0.00013	0.00067±0.00013	0.00035±0.00007	0.00072±0.00005
44	n-Decanoic acid	0.00014±0.00007	0.00012±0.00009		0.00042±0.00014	0.00025±0.00012	-0.00003±0.00003	0.00064±0.00013

Fig S2. Phenotypes of malt-aroma yogurt and control group yogurt. (A), (B) and (C) malt-aroma yogurt, (D), (E) and (F) control group yogurt. (A) and (D) viable bacteria count, (B) and (E) lactic acid, (C) and (F) pH

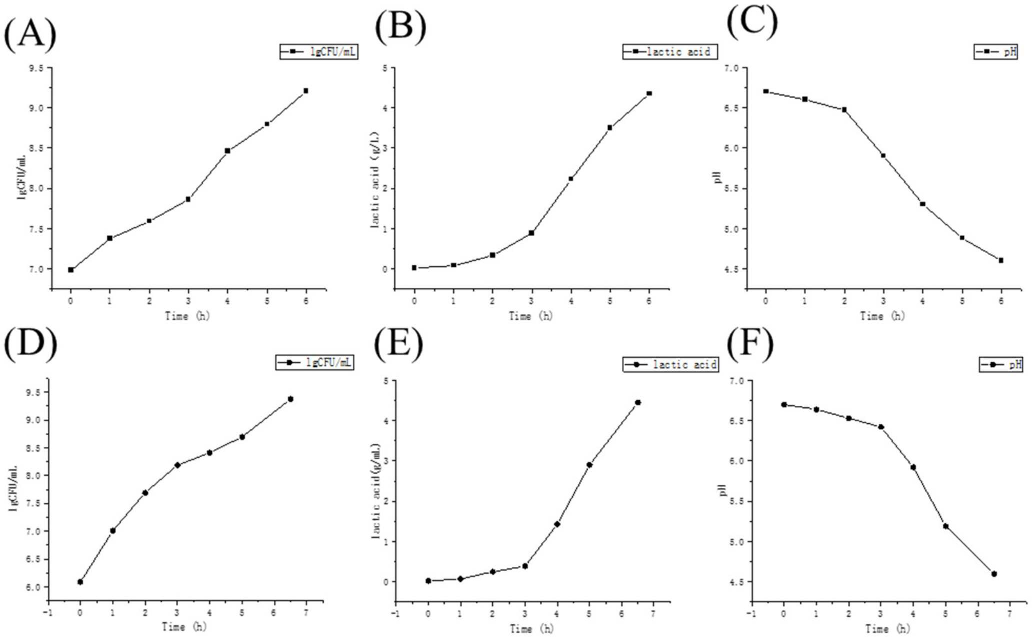

Fig S3. Heatmap of Spearman correlations between key aroma compounds and differential metabolites

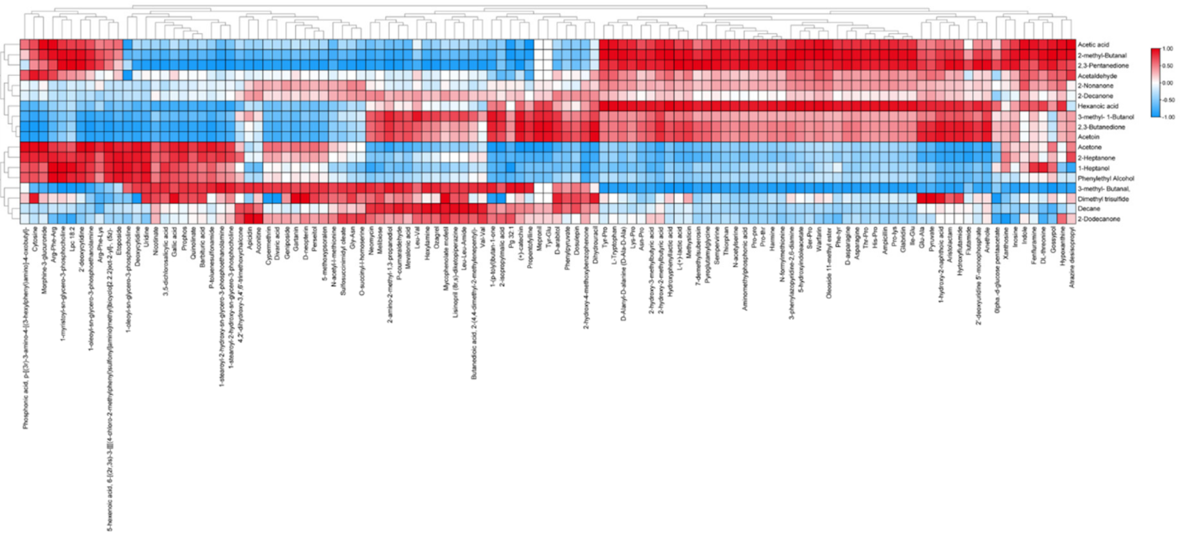

Supplement: Supplementary file 1 [file foods-15-00272-s001.zip › foods-4066968-supplementary.pdf]
